# LlpB represents a second subclass of lectin‐like bacteriocins

**DOI:** 10.1111/1751-7915.13373

**Published:** 2019-01-31

**Authors:** Maarten G. K. Ghequire, René De Mot

**Affiliations:** ^1^ Centre of Microbial and Plant Genetics KU Leuven Kasteelpark Arenberg 20 bus 2460 3001 Heverlee Belgium

## Abstract

Bacteriocins are secreted bacterial proteins that selectively kill related strains. Lectin‐like bacteriocins are atypical bacteriocins not requiring a cognate immunity factor and have been primarily studied in *Pseudomonas*. These so‐called LlpAs are composed of a tandem of B‐lectin domains. One domain interacts with d‐rhamnose residues in the common polysaccharide antigen of *Pseudomonas* lipopolysaccharide (LPS). The other lectin domain is crucial for interference with the outer membrane protein assembly machinery by interacting with surface‐exposed loops of its central component BamA. Via genome mining, we identified a second subclass of *Pseudomonas* lectin‐like proteins, termed LlpB, consisting of a single B‐lectin domain. We show that these proteins also display bactericidal activity. Among LlpB‐resistant transposon mutants of an LlpB‐susceptible *Pseudomona*s strain, a major subset was hit in an acyltransferase gene, predicted to be involved in LPS core modification, hereby suggesting that LlpBs equally attach to LPS for surface anchoring. This indicates that LPS binding and target strain specificity are condensed in a single B‐lectin domain. The identification of this second subclass of lectin‐like bacteriocins further expands the toolbox of antibacterial warfare deployed by bacteria and holds potential for their integration in biotechnological applications.

## Introduction

Bacteriocins are secreted ribosomally encoded antibacterial peptides, proteins or multi‐protein complexes that selectively kill phylogenetically related strains, thus facilitating the colonization of competitive environments. Among Gram‐negative bacteria, bacteriocins from *Escherichia coli* (colicins) and *Pseudomonas aeruginosa* (pyocins) serve as model systems for studying receptor binding, cell import mechanisms and toxin‐immunity interactions (Cascales *et al*., 2007; Papadakos *et al*., 2012; Ghequire and De Mot, 2014; Chassaing and Cascales, 2018). These compounds are potent antibacterials and their use in food and therapeutic applications is currently being investigated (Schulz *et al*., 2015; Paškevičius *et al*., 2017; Scholl, 2017; Schneider *et al*., 2018). Major advantages of bacteriocins include biodegradability, selective killing and eligibility (of some bacteriocins) for large‐scale production in plants (Behrens *et al*., 2017; Ghequire and De Mot, 2018).

To date, four main classes of *Pseudomonas* bacteriocins have been described, highly diverse in molecular architecture and killing mechanism: R‐ and F‐type tailocins (Ghequire and De Mot, 2015; Scholl, 2017), modular (or S‐type) bacteriocins (Jamet and Nassif, 2015), B‐type microcins (Metelev *et al*., 2013) and lectin‐like bacteriocins (Ghequire *et al*., 2018b). The latter set of bacteriocins (also called LlpAs) are composed of two B‐lectin domains followed by a short carboxy‐terminal extension and share structural similarity with lectins from monocot plants (Ghequire *et al*., 2013; McCaughey *et al*., 2014). The carboxy‐terminal lectin domain of these antibacterial proteins binds to d‐rhamnose (McCaughey *et al*., 2014), the major constituent of the common polysaccharide antigen in the lipopolysaccharide (LPS) layer (Lam *et al*., 2011), in contrast to B‐lectins from plants which show a much higher affinity for d‐mannose (Barre *et al*., 1996). The amino‐terminal lectin domain selectively interacts with the essential outer membrane protein BamA (Ghequire *et al*., 2018b). The latter protein acts as an insertase responsible for the integration of new proteins in the outer membrane (Noinaj *et al*., 2015). It remains unclear how LlpA interacts with the surface‐exposed loops of BamA and how cellular killing is achieved. Given the lack of a distinct toxin domain and cognate immunity factor as found in modular bacteriocins (Sharp *et al*., 2017), LlpA killing is likely initiated upon contact with the outer membrane. This way no subsequent bacteriocin import, as is the case for modular bacteriocins, would be required (White *et al*., 2017). Several other hypothetical prokaryotic proteins in which a B‐lectin domain is combined with (an)other domain(s) have been identified (Ghequire *et al*., 2012b). For a protein with an amino‐terminal B‐lectin domain fused to a putative peptidase domain, bacteriocin activity has been described: albusin B from ruminal bacterium *Ruminococcus albus* 7 kills *Ruminococcus flavefaciens* (Chen *et al*., 2004). However, how these domains contribute to bacteriocin activity has not been studied. Homologues of this bacteriocin gene are present in some other strains of this Firmicutes species (Azevedo *et al*., 2015). In *Mycobacterium smegmatis* MC^2^155, a protein consisting of a B‐lectin and a LysM domain has been described (Patra *et al*., 2011), though it remains unclear whether this compound serves a role in bacterial antagonism.

In this paper, we report on the bacteriocin activity of a distinct type of *Pseudomonas* lectin‐like protein, termed LlpB, consisting of a single B‐lectin domain and a short carboxy‐terminal extension. Characterization of transposon mutants resistant to an LlpB from a *Pseudomonas fluorescens* strain indicates that target recognition involves LPS of susceptible cells.

## Results and discussion

### LlpB: a distinct type of lectin‐like protein in *Pseudomonas*

**Figure 1:**
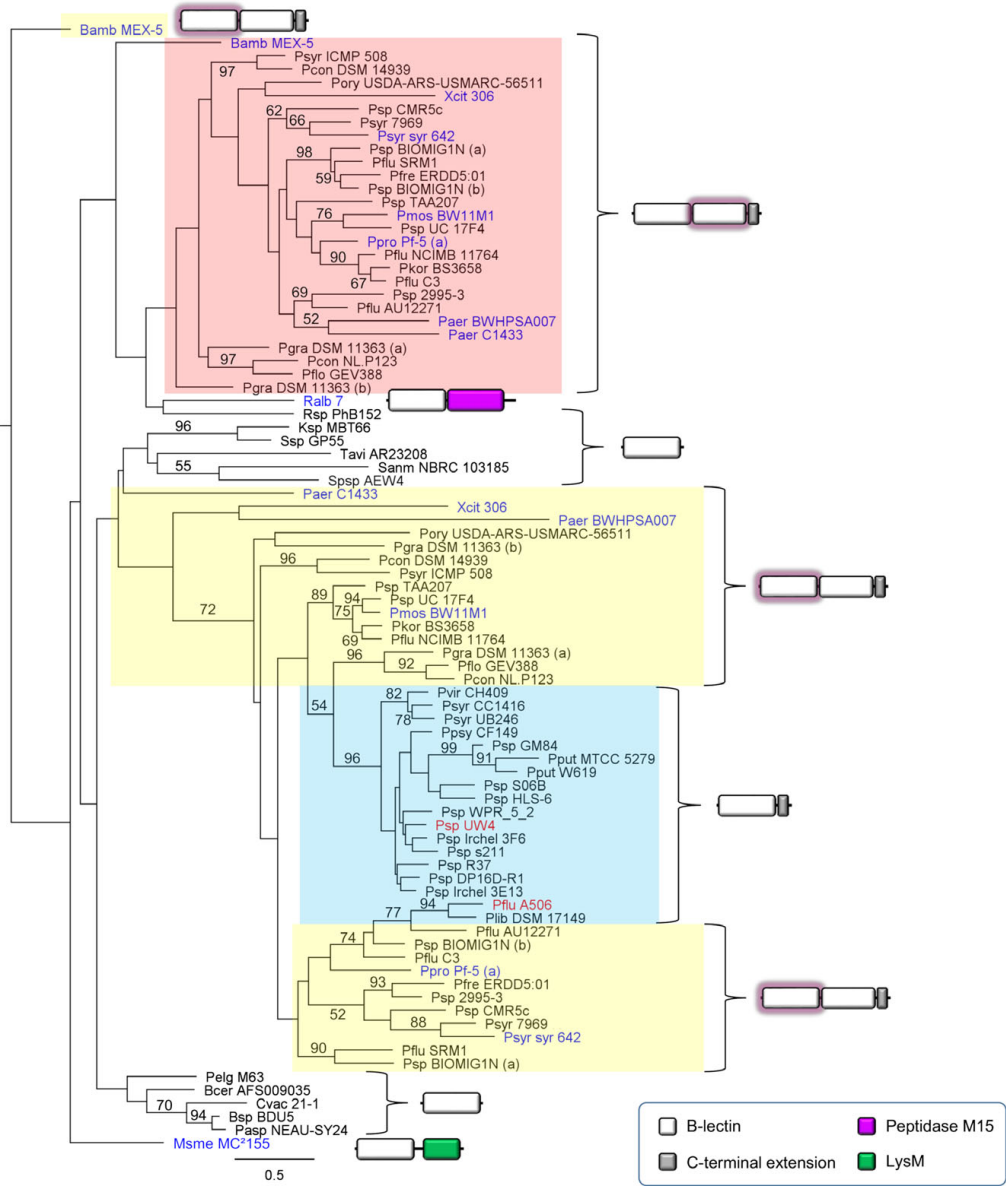
Maximum likelihood phylogenetic tree of B‐lectin domains from LlpA and LlpB proteins in *Pseudomonas*, characterized LlpA and B‐lectin domain‐containing proteins retrieved in other bacteria, and select B‐lectin mono‐domain proteins in other bacteria. The domain architecture is specified by a schematic representation, and domains are coloured according to function (see colour legend in box). Amino‐terminal and carboxy‐terminal lectin domains from LlpAs and lectin domains from LlpBs are shown on a yellow, red and blue background, respectively. B‐lectin domains from LlpBs cluster with the amino‐terminal domain of LlpAs. In the case of LlpAs, the B‐lectin domain shown in the respective cluster is highlighted by a glowing background. Highly similar sequences (> 75% pairwise amino acid sequence identity for full length LlpA/LlpB proteins) are represented by one sequence only. Previously characterized proteins with a B‐lectin domain are labelled in blue, and LlpBs characterized in this study in red. Multiple LlpA/LlpB bacteriocins in a particular strain are specified by extensions (a) and (b). Phylogenetic analysis was performed with PhyML, using the JTT substitution model. Bootstrap values (percentages of 1000 replicates) higher than 50 are shown at the branches. The tree is rooted to the amino‐terminal B‐lectin domain of the LlpA *from Burkholderia ambifaria* MEX‐5. Scale bar represents 0.5 substitutions per site. Bamb, *Burkholderia ambifaria*; Bcer, *Bacillus cereus*; Bsp, *Burkholderia* sp.; Cvac, *Chromobacterium vaccinii*; Ksp, *Kitasatospora* sp.; Msme, *Mycobacterium smegmatis*; Paer, *Pseudomonas aeruginosa*; Pasp, *Paraburkholderia* sp.; Pcon, *Pseudomonas congelans*; Pelg, *Paenibacillus elgii*; Pflo, *Pseudomonas floridensis*; Pflu, *Pseudomonas fluorescens*; Pfre, *Pseudomonas frederiksbergensis*; Pgra, *Pseudomonas graminis*; Pkor, *Pseudomonas koreensis*; Plib, *Pseudomonas libanensis*; Pmos, *Pseudomonas mosselii*; Pory, *Pseudomonas oryzihabitans*; Ppro, *Pseudomonas protegens*; Ppsy, *Pseudomonas psychrophila*; Pput, *Pseudomonas putida*; Psp, *Pseudomonas* sp.; Psyr (syr), *Pseudomonas syringae* (pathovar syringae); Pvir, *Pseudomonas viridiflava*; Ralb, *Ruminococcus albus*; Rsp, *Rathayibacter* sp.; Sanm, *Streptacidiphilus anmyonensis*; Spsp, *Sphingobium* sp.; Ssp, *Streptomyces* sp.; Tavi, *Tumebacillus avium*; Xcit, *Xanthomonas citri*.

Using proteobacterial B‐lectin modules (Pfam PF01453) of *Pseudomonas* LlpAs as search queries, BlastP homology searches previously revealed a second group of lectin‐like proteins in pseudomonads (Ghequire *et al*., 2012b; Loper *et al*., 2012; Ghequire and De Mot, 2014). These proteins (~19.8 kDa) consist of a single B‐lectin domain and a carboxy‐terminal extension of ~32 AA. The latter stretch is poorly conserved but typified by a number of basic and aromatic residues (Fig. S1), similarly to *Pseudomonas* LlpAs (Ghequire *et al*., 2013). Phylogenetic analysis shows that the predicted lectin modules of these proteins, further called LlpBs, cluster with the amino‐terminal domains of LlpAs, acting as target selectivity determinants in these bacteriocins (Ghequire *et al*., 2013). The LlpB sequences fall apart in two distinct branches, of which the smaller one is exclusively populated by representatives belonging to the *P. fluorescens* species group (Fig. 1). As seen for LlpAs, the putative sugar‐binding motifs in LlpBs display strongly differing degrees of sequence conservation, with the first and last of the tree pockets being well conserved (Fig. S1).

*llpB* genes mainly occur in plant‐ and soil‐associated *Pseudomonas* isolates, but are lacking from *P. aeruginosa* genomes. They are rather rare overall (~3.4% of assembled *Pseudomonas* genomes, excluding the *P. aeruginosa* species group), but appear relatively more frequent in *Pseudomonas syringae* (~7.5% of strains belonging to the *P. syringae* species group). Interestingly, *llpB* genes do not co‐occur with *llpA* genes within a single strain. Furthermore, quite some bacteria encode LlpB‐like proteins lacking a carboxy‐terminal extension (Fig. 1). Such mono‐B‐lectin domain proteins, often preceded by a (predicted) SecA secretion signal sequence (http://www.compgen.org/tools/PRED-TAT) , are for example found in Actinobacteria (e.g. *Kitasatospora*,* Streptacidiphilus* and *Streptomyces*) and Firmicutes (e.g. *Brevibacillus* and *Paenibacillus*). As seen for LlpA‐encoding *Pseudomonas* strains (Parret *et al*., 2005; Ghequire *et al*., 2018b), isolates may host (up to) two *llpB* genes, for example *Pseudomonas* sp. FW104‐R4. If so, *llpB* genes are organized in tandem, whereas *llpA* genes in strains carrying two representatives usually appear at distant loci (Ghequire and De Mot, 2014). As noted for other (mid‐sized) bacteriocins (Ghequire *et al*., 2015, 2017a,2017b; Dingemans *et al*., 2016; Sharp *et al*., 2017), *llpB* genes are typified by a lower GC content than the genomic average (~47% versus ~60%), pointing towards foreign origin. Yet another similarity with *llpA* genes is that some of these *llpB* genes arise in prophage/tailocin clusters (Ghequire *et al*., 2015), for example in a Rp3 tailocin gene cluster of *Pseudomonas libanensis* DSM 17149. Such association is confined to the minor branch of *llpB‐*carrying isolates (Fig. 1). In contrast, in the large clade they mainly occur at two other loci: downstream of sulphate adenylyltransferase *cysN* or downstream of a flavin monoamine oxidase gene (data not shown). In some strains, a modular bacteriocin‐immunity gene tandem is integrated between *cysN* and *llpB*, for example in *P. putida* MTCC 5279 (putative HNH DNase toxin), underlining the plasticity of the locus. Taken together, the striking parallels of LlpBs with other bacteriocins suggest that these proteins may also exert an antibacterial function. To explore this further, representative and divergent LlpBs (Fig. 1) from biocontrol strain *P. fluorescens* A506 (Loper *et al*., 2012) and plant growth‐promoting rhizobacterium *Pseudomonas* sp. UW4 (Duan *et al*., 2013) were selected for further characterization.

### Bacteriocin activity of LlpBs

**Figure 2:**
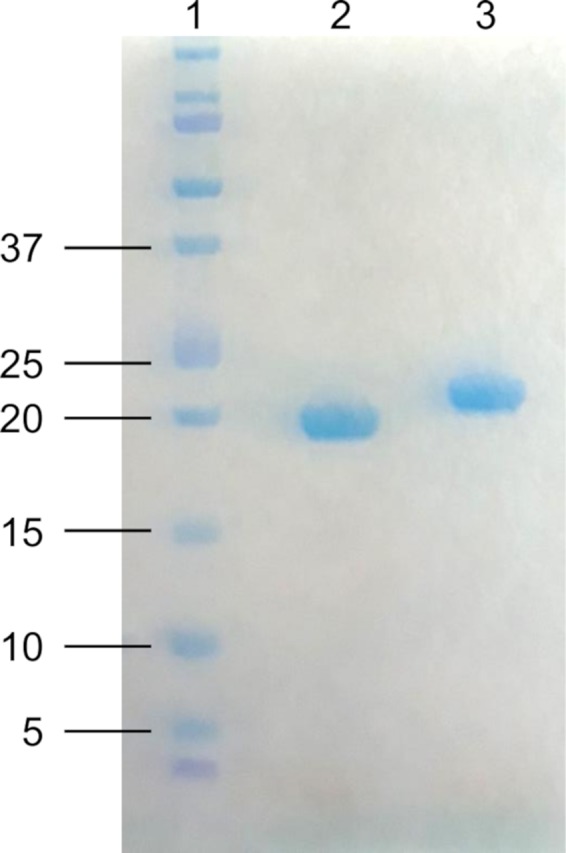
SDS‐PAGE electrophoresis of purified recombinant LlpB proteins from strains *P. fluorescens* A506 and *Pseudomonas* sp. UW4. Lane 1, Precision Plus Dual Xtra size marker (kDa); lane 2, LlpB_P__fluA506_ (~19 kDa, predicted size 20.6 kDa); lane 3, LlpB_P__sp_ _UW__4_ (~21 kDa, predicted size 21.4 kDa).

*llpB* genes from strains A506 (locus_tag PflA506_2041) and UW4 (locus_tag PPUTUW4_RS25815, codon‐optimized) were PCR‐amplified, digested and cloned in pET28a to encode an amino‐terminal His_6_‐tagged protein (primers in Table S1), resulting in pCMPG6205 and pCMPG6207, respectively. Sequence‐verified plasmids (GATC Biotech, Constance, Germany) were transformed to *E. coli* BL21(DE3). Cells grown in 500‐ml LB erlenmeyers were induced with isopropyl‐β‐d‐thiogalactopyranoside and incubated overnight, as described earlier (Ghequire *et al*., 2012b). After, cells were harvested, dissolved and sonicated, and soluble proteins isolated via centrifugation. His‐tagged proteins were purified via affinity chromatography on Ni‐NTA agarose. The presence of recombinant protein in the imidazole‐eluted fractions was confirmed via SDS‐PAGE, and samples were further polished by gel filtration. The calculated molecular weights of His_6_‐tagged LlpBs (20.6 kDa LlpB_PfluA506_; 21.4 kDa LlpB_PspUW4_) match well with the apparent sizes of the recombinant proteins as estimated by SDS‐PAGE (Fig. 2).

Antagonistic activity of the LlpBs was evaluated via spot assay against a panel of pseudomonads, including several *Pseudomonas* reference strains. Ten‐μl drops of recombinant protein (concentration 1 mg ml^−1^) were applied onto bacterial cell lawns, incubated overnight, and scored for the presence of zones of growth inhibition the following day (Hockett and Baltrus, 2017). For both LlpBs, eight out of 49 strains in the *Pseudomonas* test panel proved susceptible (Table 1), confirming the bactericidal function of these proteins. Five strains were killed by both LlpBs, despite their low sequence identity (~34%). As seen for other *Pseudomonas* bacteriocins (LlpAs and other (non‐*P. aeruginosa*) bacteriocins) (Ghequire *et al*., 2012a, 2015), LlpB activity surpasses species boundaries: the bacteriocins from *P. fluorescens* A506 and *Pseudomonas* sp. UW4 [*P. jesseni* group (Gomila *et al*., 2015; Garrido‐Sanz *et al*., 2016)] both also kill strains from the *P. stutzeri* and *P. syringae* groups.

**Table 1:** Antibacterial activity of purified recombinant LlpBs against *Pseudomonas* isolates

Indicator strain	Growth inhibition^a^ by	
	LlpB_PfluA506_	LlpB_PspUW4_
*P. aeruginosa* group		
*P. aeruginosa* LMG 1242	−	−
*P. aeruginosa* ATCC27853	−	−
*P. aeruginosa* PAO1	−	−
*P. aeruginosa* UCBPP‐PA14	−	−
*P. resinovorans* LMG 2274	−	−
*P. fluorescens* complex		
*P. chlororaphis* subsp. aureofaciens LMG 1245	−	−
*P. chlororaphis* subsp. chlororaphis LMG 5004	−	−
*P. fluorescens* 2‐79	−	−
*P. fluorescens* 13‐79	+	−
*P. fluorescens* A1‐B	−	−
*P. fluorescens* CC‐848406‐E	−	−
*P. fluorescens* F113	−	−
*P. fluorescens* LMG 1794	+	−
*P. fluorescens* LMG 2210	−	−
*P. fluorescens* OE 39.4	−	−
*P. fluorescens* OE 48.2	−	−
*P. fluorescens* Pf0‐1	−	−
*P. fluorescens* PGSB 7705	+	T
*P. fluorescens* PGSB 7716	−	−
*P. fluorescens* PGSB 7947	−	−
*P. fluorescens* PGSB 8301	−	+
*P. fluorescens* PGSB 8472	−	−
*P. fluorescens* SBW25	−	−
*P. fluorescens* WCS141	−	−
*P. fluorescens* WCS365	−	T
*P. protegens* CHA0^b^	−	−
*P. tolaasii* CH36	−	−
*P. tolaasii* LMG 2342	−	−
*P. tolaasii* LMG 2344	−	−
*P. putida* group		
*P. putida* KT2440	−	−
*P. putida* LMG 2257	−	−
*P. putida* OE 53.2	−	−
*P. putida* WCS358	−	−
*P. stutzeri* group		
*P. stutzeri* LMG 11199	−	−
*P. stutzeri* LMG 1228	+	+
*P. syringae* group		
*P. cichorii* LMG 2162	−	T
*P. savastanoi* LMG 2209	−	−
*P. savastanoi* LMG 5154	−	−
*P. savastanoi* LMG 5485	+	−
*P. savastanoi* LMG 6768	−	−
*P. savastanoi* LMG 17581	−	−
*P. syringae* GR12‐2R3	+	+
*P. syringae* pv. glycinea LMG 5066	+	+
*P. syringae* pv. syringae LMG 1247	−	−
*P. syringae* pv. tabaci LMG 5192	−	−
*P. syringae* pv. tomato DC3000	−	−
*P. viridiflava* LMG 2352	+	+
Other *Pseudomonas* spp.		
*P. agarici* LMG 2112	−	−
*P. mendocina* LMG 1223	−	−

**a.** Growth inhibition due to LlpB bacteriocin activity was scored as follows: +, clear halo; T, turbid halo; −, no zone of growth inhibition. Running buffer was used as a negative control.

**b**. Of the strains used in the test panel (and for which genome sequence information is available), only *P. protegens* CHA0 carries an *llpA* gene in its genome. No strain contains an *llpB* gene.

### Genes affected in LlpB‐resistant mutants indicate a key role of LPS in target cell susceptibility

The first and last of the three sugar‐binding motifs in LlpBs show sequence similarity with the consensus motif accounting for d‐mannose binding in plant lectins, QxDxNxVxY (Ghequire *et al*., 2012b). Given the role assigned to d‐rhamnose as a ligand for LlpAs, we hypothesized that one or both of these lectin motifs in LlpBs may bind to carbohydrates from lipopolysaccharides as well, enabling target cell attachment in a similar way.

**Figure 3:**
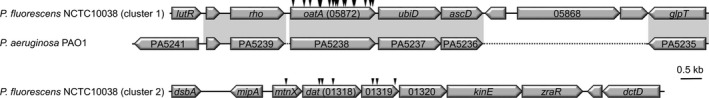
Schematic gene representation of two genomic regions in *Pseudomonas fluorescens* LMG 1794^T^ (NCTC10038^T^) susceptible to bacteriocin LlpB_P__fluA506_. Genes are shown as arrows and insert locations of transposon pRL27 are indicated with black triangles. Gene synteny of the locus of *oatA*, target of the large majority of the LlpB‐resistant mutants, with the corresponding region in reference strain *P. aeruginosa* PAO1 is shown by grey shading. Dotted lines indicate the lack of an equivalent region.

In search for susceptibility determinants of LlpB killing, a mutant library was created in *P. fluorescens* LMG 1794^T^ (sequenced as NCTC10038^T^) using transposon delivery vector pRL27 (Larsen *et al*., 2002), via triparental conjugation. Transposon mutants were pooled, supplemented with concentrated LlpB_PfluA506_ (~5 mg ml^−1^), and subsequently plated. Following day, colonies were selected, verified for bacteriocin resistance and transposon insertion sites determined, as described earlier (Ghequire *et al*., 2017b). Interestingly, of the 34 (independent) LlpB‐resistant mutants isolated, 24 were hit in an acyltransferase gene *oatA* (NCTC10038_05872) (Fig. 3). The encoded protein shares 27% amino acid identity with *oafA*, previously studied in *Salmonella* Typhimurium and shown to function as an *O*‐antigen acetylase (Slauch *et al*., 1996). Gene synteny and significant sequence similarity (48% pairwise amino acid identity) can be noted for *PA5238* from *Pseudomonas aeruginosa* PAO1. Lipopolysaccharide acetylation activity has been proposed for the latter enzyme (King *et al*., 2009), but remains to be verified. The repeating units constituting the O‐specific polysaccharide chains of LPS in *P. fluorescens* LMG 1794 have been determined and consist of l‐rhamnose and *N*‐acetyl‐d‐fucose (Veremecheĭnko *et al*., 2005). Given that *PA5238* was suggested to play a role in *O*‐acetylation of the LPS core and not of the repeating units (King *et al*., 2009), we thus do not expect these two carbohydrate residues to interact with LlpB_PfluA506_. It remains to be assessed whether other LlpBs equally depend for killing on the activity of this acyltransferase gene in target cells, which would be expected if these lectin‐like bacteriocins share a common LPS moiety as receptor. It should be emphasized that polar effects on the two genes downstream of NCTC10038_05872 cannot be excluded *a priori*, though the multiple transposon insertions independently hitting *oatA* argue against this. When evaluating our strain panel for the presence of *oatA* and *oatA*‐like genes, we found that the majority of the strains (for which a full or draft genome is available, 23/30) encodes such an acyltransferase, including all the strains killed by one or both of the LlpBs.

A second set of seven transposon mutants were hit in an operon that is possibly involved in LPS biogenesis as well (Fig. 3). This cluster is conserved in *Pseudomonas* species, but apparently lacks from *P. aeruginosa* genomes. In LPS of *P. fluorescens* NCTC10038, different amino sugars have been detected (Wilkinson, 1972), which may require *dat* aminotransferase activity. Whether this second cluster plays a role in LPS biosynthesis remains speculative however. In the nearby future, chemical characterization of LPS constituents of different mutants obtained in this study will shed further light on the carbohydrates interacting with LlpB. Whether BamA or (an)other outer membrane protein(s) contribute to LlpB killing also remains to be investigated.

## Conflict of interest

None declared.

## Supporting information


**Fig. S1**. Multiple sequence alignment of LlpBs included in Figure 1.

**Table S1**. Primers used in this study.
