# Supplementary figures and images for: LlpB represents a second subclass of lectin‐like bacteriocins
Source: Microb Biotechnol. 2019 Jan 31;12(3):567–73. doi: 10.1111/1751-7915.13373 (PMC6465234; doi:10.1111/1751-7915.13373)

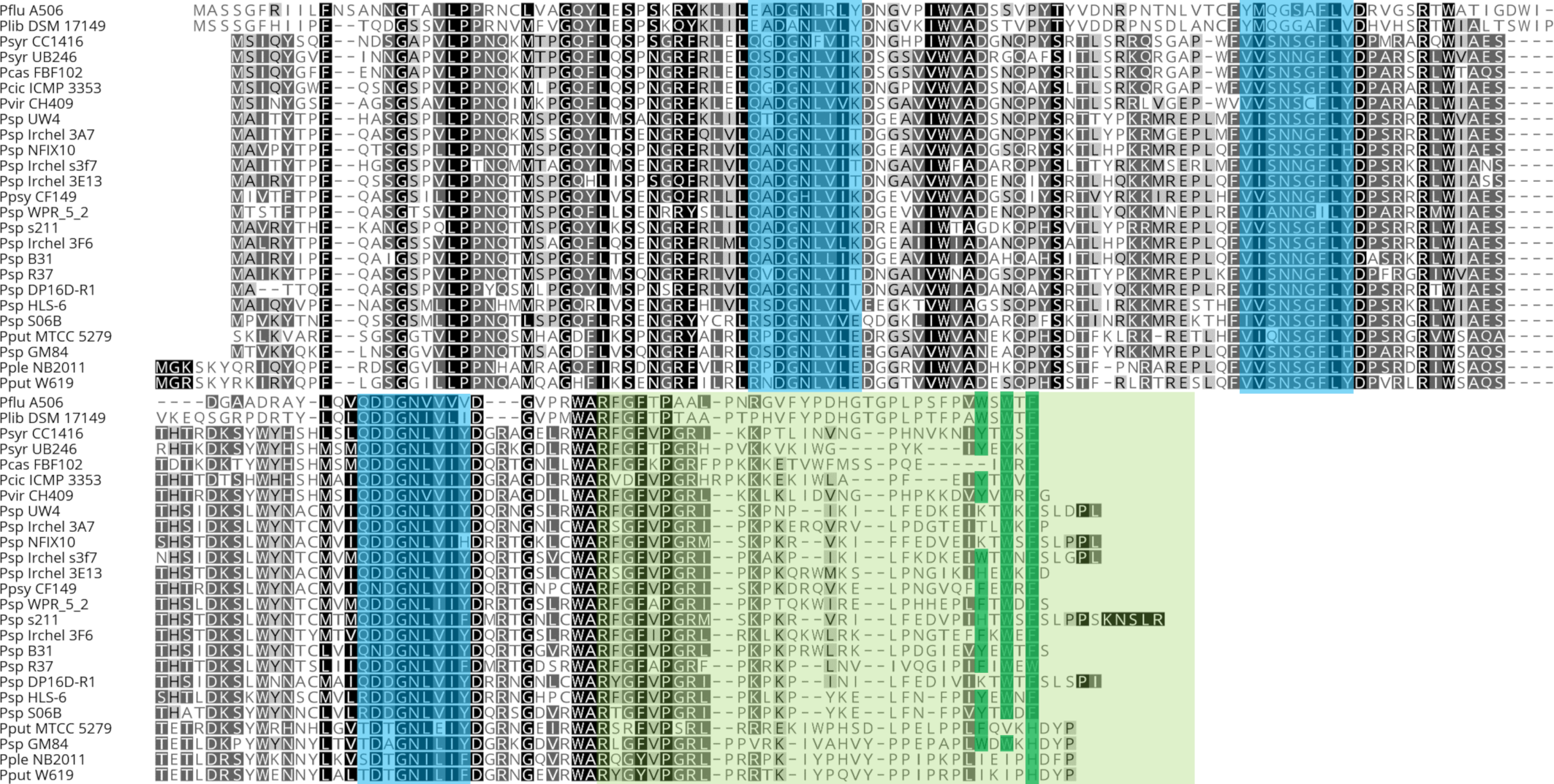

Supplement: Supplementary file 1 — Fig. S1. Multiple sequence alignment of LlpBs included in Figure 1. [file MBT2-12-567-s001.tif]
