# Supplementary material for: LlpB represents a second subclass of lectin‐like bacteriocins
Source: Microb Biotechnol. 2019 Jan 31;12(3):567–73. doi: 10.1111/1751-7915.13373 (PMC6465234; doi:10.1111/1751-7915.13373)
Supplement: Supplementary file 2 — Table S1. Primers used in this study. [file MBT2-12-567-s002.docx]

Table S1. Primers used in this study.

| **Primer number** | **Sequence^a^ (5’-3’)** | **Purpose of use** |
| --- | --- | --- |
| PGPRB-10124 | TGGCTACATATGGCTAGTAGTGGCTTTCGTATT | Cloning of *llpB_PfluA506_* |
| PGPRB-10125 | TGGCTACTCGAGTTAGAAAGTCCAGCTCCATACCG | Cloning of *llpB_PfluA506_* |
| PGPRB-10126 | TGGCTACATATGGCGATTACCTATACTCCCTTCCA | Cloning of *llpB_PspUW4_* |
| PGPRB-10127 | TGGCTACTCGAGTCAGAGCGGGTCCAGGCT | Cloning of *llpB_PspUW4_* |
| PGPRB-10249 | TGGCAGCAGCCAACTCAGCTT | Sequence validation of inserts in pET28a |
| PGPRB-10250 | TATAGGCGCCAGCAACCGCA | Sequence validation of inserts in pET28a |
| PGPRB-10273 | AACAAGCCAGGGATGTAACG | Sequencing of transposon inserts |
| PGPRB-10274 | CAGCAACACCTTCTTCACGA | Sequencing of transposon inserts |

^a^ Restriction sites incorporated in the primers are underlined: CATATG, NdeI; CTCGAG, XhoI.
