# Supplementary material for: LlpB represents a second subclass of lectin‐like bacteriocins
Source: Microb Biotechnol. 2019 Jan 31;12(3):567–73. doi: 10.1111/1751-7915.13373 (PMC6465234; doi:10.1111/1751-7915.13373)

Supporting Information for

LlpB represents a second subclass of lectin-like bacteriocins

Maarten G. K. Ghequire*, René De Mot

Centre of Microbial and Plant Genetics, KU Leuven, Kasteelpark Arenberg 20 bus 2460, 3001 Heverlee, Belgium

* Corresponding author: [maarten.ghequire@biw.kuleuven.be](mailto:maarten.ghequire@biw.kuleuven.be); Tel: +32 16 376638, Fax: +32 16 321963

Table S1. Primers used in this study.

| **Primer number** | **Sequence^a^ (5’-3’)** | **Purpose of use** |
| --- | --- | --- |
| PGPRB-10124 | TGGCTACATATGGCTAGTAGTGGCTTTCGTATT | Cloning of *llpB_PfluA506_* |
| PGPRB-10125 | TGGCTACTCGAGTTAGAAAGTCCAGCTCCATACCG | Cloning of *llpB_PfluA506_* |
| PGPRB-10126 | TGGCTACATATGGCGATTACCTATACTCCCTTCCA | Cloning of *llpB_PspUW4_* |
| PGPRB-10127 | TGGCTACTCGAGTCAGAGCGGGTCCAGGCT | Cloning of *llpB_PspUW4_* |
| PGPRB-10249 | TGGCAGCAGCCAACTCAGCTT | Sequence validation of inserts in pET28a |
| PGPRB-10250 | TATAGGCGCCAGCAACCGCA | Sequence validation of inserts in pET28a |
| PGPRB-10273 | AACAAGCCAGGGATGTAACG | Sequencing of transposon inserts |
| PGPRB-10274 | CAGCAACACCTTCTTCACGA | Sequencing of transposon inserts |

^a^ Restriction sites incorporated in the primers are underlined: CATATG, NdeI; CTCGAG, XhoI.

Figure S1. Multiple sequence alignment of LlpBs included in Figure 1. Sequence alignment was performed with MUSCLE. Highly similar LlpBs (>80% pairwise amino acid sequence identity for full length proteins) are represented by a single sequence. Carboxy-terminal sequences are highlighted in light green and positions occupied by aromatic residues at the carboxy-terminal end of most LlpBs are in dark green boxes. The (three) sugar-binding motifs in the lectin domain of LlpBs (resembling consensus QxDxNxVxY) are boxed in blue. Residue differential shading in grey reflects the degree of sequence conservation. Abbreviations as in Figure 1. Other abbreviations: Pcas, Pseudomonas caspiana; Pcic, Pseudomonas cichorii; Pple, Pseudomonas plecoglossicida.


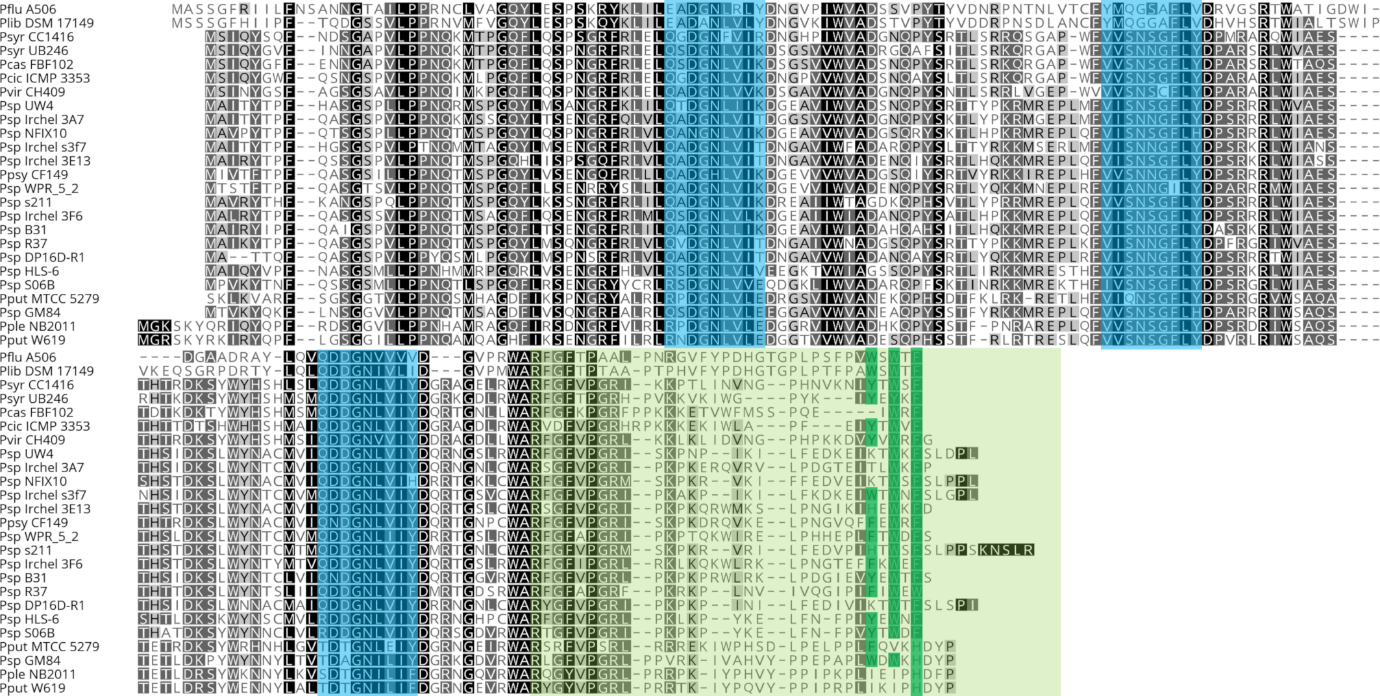

Supplement: Supplementary file 3 [file MBT2-12-567-s003.docx]
